# Intravenous N-Acetylcysteine for Prevention of Contrast-Induced Nephropathy: A Meta-Analysis of Randomized, Controlled Trials

**DOI:** 10.1371/journal.pone.0055124

**Published:** 2013-01-30

**Authors:** Zikai Sun, Qiang Fu, Longxing Cao, Wen Jin, LingLing Cheng, Zhiliang Li

**Affiliations:** 1 Cardiovascular Department, Zhujiang Hospital, Southern Medical University, Guangdong Province, China; 2 Cardiovascular Department, Guangdong No.2 Provincial People’s Hospital, Guangdong, China; University of Virginia Health System, United States of America

## Abstract

**Background:**

Contrast-induced nephropathy (CIN) is one of the common causes of acute renal insufficiency after contrast procedures. Whether intravenous N-acetylcysteine (NAC) is beneficial for the prevention of contrast-induced nephropathy is uncertain. In this meta-analysis of randomized controlled trials, we aimed to assess the efficacy of intravenous NAC for preventing CIN after administration of intravenous contrast media.

**Study Design:**

Relevant studies published up to September 2012 that investigated the efficacy of intravenous N-acetylcysteine for preventing CIN were collected from MEDLINE, OVID, EMBASE, Web of Science, Cochrane Central Register of Controlled Trials, and the conference proceedings from major cardiology and nephrology meetings. The primary outcome was CIN. Secondary outcomes included renal failure requiring dialysis, mortality, and length of hospitalization. Data were combined using random-effects models with the performance of standard tests to assess for heterogeneity and publication bias. Meta-regression analyses were also performed.

**Results:**

Ten trials involving 1916 patients met our inclusion criteria. Trials varied in patient demographic characteristics, inclusion criteria, dosing regimens, and trial quality. The summary risk ratio for contrast-induced nephropathy was 0.68 (95% CI, 0.46 to 1.02), a nonsignificant trend towards benefit in patients treated with intravenous NAC. There was evidence of significant heterogeneity in NAC effect across studies (Q = 17.42, P = 0.04; I^2^ = 48%). Meta-regression revealed no significant relation between the relative risk of CIN and identified differences in participant or study characteristics.

**Conclusion:**

This meta-analysis showed that research on intravenous N-acetylcysteine and the incidence of CIN is too inconsistent at present to warrant a conclusion on efficacy. A large, well designed trial that incorporates the evaluation of clinically relevant outcomes in participants with different underlying risks of CIN is required to more adequately assess the role for intravenous NAC in CIN prevention.

## Introduction

The increasing use of contrast media (CM) for a multitude of radiological procedures, particularly during coronary angiography, has raised concerns about the increasing incidence of a potential complication known as contrast-induced nephropathy (CIN) [1]. In patients undergoing coronary angiography, the incidence of CIN varies widely (2%–50%), with baseline presence of chronic renal disease (CRD) and diabetes mellitus being the most important risk factors [1], [2]. As the third leading cause of hospital-acquired acute renal failure [3], CIN is associated with adverse clinical outcomes, prolonged hospitalization, and increased health care costs [4]–[6]. The pathophysiology of contrast-induced nephropathy remains incompletely understood. It is hypothesized that renal vasoconstriction leading to renal medullary ischemia and direct toxicity to the kidney tubules mediated via reactive oxygen species may cause CIN [1], [7].

N-acetylcysteine (NAC) is a direct scavenger of free radicals, improves blood flow through nitric oxide–mediated pathways resulting in vasodilation, and is a precursor for the synthesis of glutathione [8]. The antioxidant and vasodilatory properties of NAC are thought to provide protection against RCIN. Results of the initial trial [9] of oral NAC for the prevention of CIN were impressive, but subsequent studies and meta-analyses performed with the data gathered by these studies have shown that the efficacy of orally-administered NAC for CIN prevention has remained unresolved to date [10].

Bioavailability of oral NAC is low, ranging from 4% to 10%, as a result of first-pass hepatic metabolism [11], [12], suggests that only a small proportion of the administered dose is available for renal protection [13], [14]. Given the considerably different pharmacodynamic and pharmacokinetic profiles between intravenous and oral NAC, it has been suggested that the intravenous form of NAC may be more effective in preventing CIN [15]. However, similar to the trials of orally administered NAC, trials with the intravenous formulation have shown mixed results [16]–[25]; a few studies demonstrated a reduction in incidence of CIN [16]–[18] while others reported a no significant benefit [19]–[25]. We therefore performed a meta-analysis of the randomized controlled trials (RCTs) in order to evaluate the efficacy of intravenous NAC for the prevention of contrast-induced nephropathy and to assess the magnitude of any such effect.

## Materials and Methods

### Search Strategy

The overview of RCTs was conducted in accordance with the Preferred Reporting Items for Systematic Reviews and Meta-analysis (PRISMA) statement [26]. We conducted a systematic literature search of MEDLINE (1966 – September 2012), OVID (1966 – September 2012), EMBASE (1966 – September 2012), Web of Science (1997 – September 2012) and the Cochrane Central Register of Controlled Trials (1996 – September 2012) for all relevant articles. We derived three comprehensive search themes that were then combined using the Boolean operator “AND”. For the theme “contrast media”, we used combinations of MeSH, entry terms and text words: contrast, radiocontrast, contrast medium, contrast media, contrast dye, radiocontrast media, radiocontrast medium and contrast agent. For the theme “renal insufficiency”, we used: renal insufficiency, renal failure, diabetic nephropathies, nephritis, nephropathy, nephrotoxic, contrast-induced nephropathy and contrast-associated nephropathy. For the theme “intravenous NAC”, N-acetylcysteine, NAC, acetylcysteine and Acetadote were used. We did not restrict by language or type of article. Abstract lists from the 2006 and 2011 scientific meetings of the American Heart Association, the American College of Cardiology, Society of Interventional Radiology, and the American Society of Nephrology were also searched for relevant reports. References of published articles were examined to identify other potentially relevant studies. Both the investigators independently reviewed all relevant articles, with discrepancies resolved by consensus. Abstracts were not considered if they represented partial or complete results of a later published full-text article.

### Selection Criteria

Studies were limited to prospective, randomized, controlled trials (PRCTs) investigating the efficacy of intravenous NAC in preventing CIN, in which at least one of the treatment groups received NAC, administered intravenously, immediately before, during, or immediately after contrast exposure at any dose, for any length of time. We required that individual studies reported sufficient data of the primary outcome for construction of a two-by-two table. Studies with no cases of contrast-induced nephropathy were observed in either the treatment or control group were excluded from our meta-analyses. Trials that were retrospective, non-randomized or compared different preventive measures without placebo control group were prospectively excluded from further analysis. Quasi-randomized trials (in which the methods of allocating participants to a treatment were not strictly random, such as by date of birth, hospital record number or weekday of admission) were excluded. Studies combined oral and intravenous NAC preparations were excluded from this analysis. Studies were not limited to trials involving patients with chronic renal insufficiency only.

### Data Extraction

Two reviewers (LXC and WJ) independently reviewed studies identified by the described search strategy to determine eligibility and perform data abstraction using standardized data collection forms. The following information was sought from each article: patient characteristics (mean age, proportion of men, baseline creatinine, and patients with diabetes mellitus), type of radiologic or angiographic imaging, inclusion and exclusion criteria, type and dose of contrast media used, hydration protocol, specific definition of contrast-related nephropathy, dose of N-acetylcysteine and timing of N-acetylcysteine administration. Attempts were made to contact authors of included studies in order to clarify or collect additional data. Trials that still lacked outcome data necessary for planned analyses were excluded.

### Outcome

The primary outcome of interest was the development of CIN defined as a rise in creatinine level of either at least 0.5 mg/dL or 25% above baseline after the exposure to contrast medium. Secondary outcomes included mortality, need for dialysis, and length of hospitalization. In case of trials in which the incidence was reported at 48 hours and other time periods, the 48-hour incidence was given precedence because this is the most common time point for ascertaining contrast-induced nephropathy [27].

### Assessment of Methodological Quality

Two reviewers (QF and LLC) independently assessed methodological quality of individual studies. For studies in which the random allocation sequence was unclear, [16], [18], [23], [25] we attempted to contact authors for clarification. Quality assessment was judged on concealment of treatment allocation, similarity of study groups at baseline, eligibility criteria, use of a placebo, use of any blinding procedure, reporting of losses to follow-up, and intention-to-treat analysis [28]. An overall quality score was determined for each study as described by Jadad et al [29]. Each PRCT included in the analysis scored at least 1 on the five-point scale, with higher scores indicating greater trial quality. Any disagreements in abstracted data between the reviewers were adjudicated by a third reviewer (ZL).

### Assessment of Heterogeneity

The presence of heterogeneity across studies was evaluated using both the Cochrane’s Q and I^2^ statistics. The Q statistic was calculated to assess if significant heterogeneity was present between the included trials. Since the Q statistic indicated that significant heterogeneity (p<0.10 for Q) was present, we used the random-effects model to combine the effect sizes of the included studies. An I^2^ value, which range from 0% to 100%, represents the percentage of total variation across studies due to heterogeneity rather than chance [30]. A value of 0% indicates no observed heterogeneity. Higgins et al. [31] suggest describing I^2^ values of 25%, 50%, and 75% as low, moderate, and high, respectively.

### Data Synthesis and Statistical Analysis

Data from all of the selected randomized controlled trials were combined to estimate the pooled risk ratio (RR) with 95% confidence intervals (CIs) using a random-effects model as described by Der Simonian and Laird [32]. We performed random meta-regression analyses to assess the association between RR estimates from the trials and characteristics of trials and their participating patients [33]. All study characteristics were selected a priori as potentially influential. The small number of trials precluded the use of multivariable meta-regression. Selected study characteristics were mean age, volume of contrast media administered, total NAC dose, baseline SCr level, proportion with diabetes, study size, publication date, and specific study quality factors. The method used to estimate the between study variance was the restricted maximum-likelihood (REML). A visual inspection of funnel plots and Egger’s weighted regression statistics were used to assess the presence of publication bias [34], [35]. A p value of less than 0.05 was judged significant with the exception of the Q statistics, in which a significance level of less than 0.1 was chosen. All statistical analyses were performed with Stata version 10.0 (StataCorp, College Station, TX).

## Results

### Study Selection

**Figure 1:**
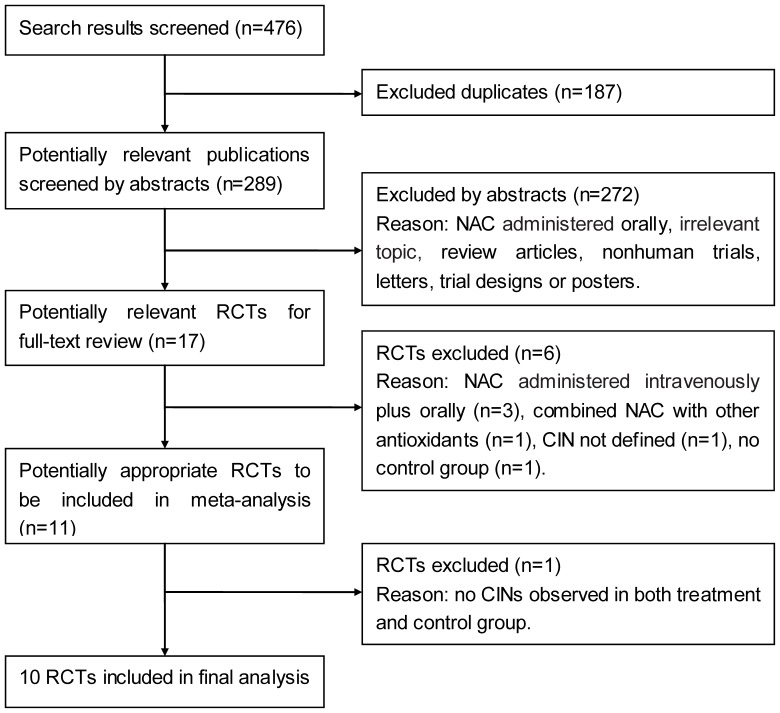
Flow diagram of selection process. Abbreviations: RCT = randomized controlled trial.

A flow chart summarizing search results is provided in Figure 1. Our initial search yielded 476 citations. We excluded 187 of these by title search due to duplicate publications. Then the titles and abstracts of the remaining 289 articles were reviewed. Of these, 272 articles were excluded, with the most common reasons for exclusion being the intervention of using NAC administered orally, or the assessment of non–nephropathy-related outcomes or use of nonhuman specimens, leaving 17 articles full articles for full publication review. The full articles were then reviewed, and a further 6 articles were excluded because the studies used combined oral and intravenous NAC preparations (n = 3) [36]–[38], study compared intravenous NAC plus intravenous sodium bicarbonate to hydration alone (n = 1) [39], CIN was not defined in the study (n = 1) [40], and study did not include a control group(n = 1) [41], One of the remaining 11 articles was excluded from our meta-analyses because no cases of contrast-induced acute kidney injury were observed in either the treatment or control group, and without other needed clinical endpoints reported [42]. Thus, the final analysis included 10 studies fulfilled our inclusion criteria [16]–[25].

### Study and Patient Characteristics

The pooled baseline clinical characteristics of the study patients are displayed in Table S1. The 10 RCTs included a total of 1914 patients (range 80–447), randomly assigned to NAC (n = 962) vs. control (n = 954) groups. All studies were performed in patients undergoing cardiac catheterization or peripheral angiography, except for the study by Poletti et al. [23], which was performed in patients undergoing computed tomography. Of the 10 trials, one trial evaluated the efficacy of N-acetylcysteine in patients with normal kidney function, [22] five trials evaluated patients with chronic kidney disease (CKD) [16]–[18], [21], [23], and the other four trials evaluated patients with both normal renal function and CKD [19], [20], [24], [25]. Patients with diabetes mellitus were included in all studies, with the prevalence varying between 12.5% and 46.9%.

The definition of CIN was variable across studies. One study defined CIN as a ≥44.2 µmol/L increase in serum creatinine from baseline [21], four used a ≥25% increase in serum creatinine from baseline [16], [23]–[25], five used either a ≥44.2 µmol/L or a ≥25% increase in serum creatinine from baseline [17]–[20], [22].

Table S1 also describes the protocols for the administration of NAC as well as the regimen of intravenous fluid hydration. Studies varied widely in their dosing regimen for NAC. Most of the trials studied similar dosing regimens as in the oral NAC trials: 500–1200 mg once or twice daily. No individual dose of intravenous NAC was less than 500 mg. However, in the first study of IV NAC treatment (RAPPID) [16], a substantially higher amount of NAC was administered 150 mg/kg over 30 minutes, followed by 50 mg/kg over 4 hours. In this study, the average dose of intravenous NAC was approximately 14 g for one person. Of the 10 studies, all patients were administered a hydration protocol around their procedure and all received low or iso-osmolar non-ionic contrast media, but the total amount of saline given was not consistently reported and the dose of contrast agent varied widely. The lowest average dose of contrast agent reported was 120 ml, and the highest dose was 238 ml.

### Assessment of Methodological Quality

Quality characteristics of each study are displayed in Table S2. All of the studies included patients with similar baseline characteristics. Seven of the 10 studies described the Randomization process. Participants in eight studies received a placebo. Six of the 10 studies reported blinding of both patients and providers to treatment assignment. Concealment of allocation and the intention to treat analysis were not provided in most studies.

### Contrast-induced Nephropathy

The reported incidence of contrast-induced nephropathy was variable across studies. The incidence of CIN in the control group ranged from 5.9% to 23.8% with an average of 14.3%. The incidence of CIN in the treatment group ranged from 2.5% to 16.0% with an average of 7.9%. Three studies provided evidence of a risk reduction for development of CIN with NAC [16]–[18], whereas seven studies reported no evidence of benefit [19]–[25].

**Figure 2:**
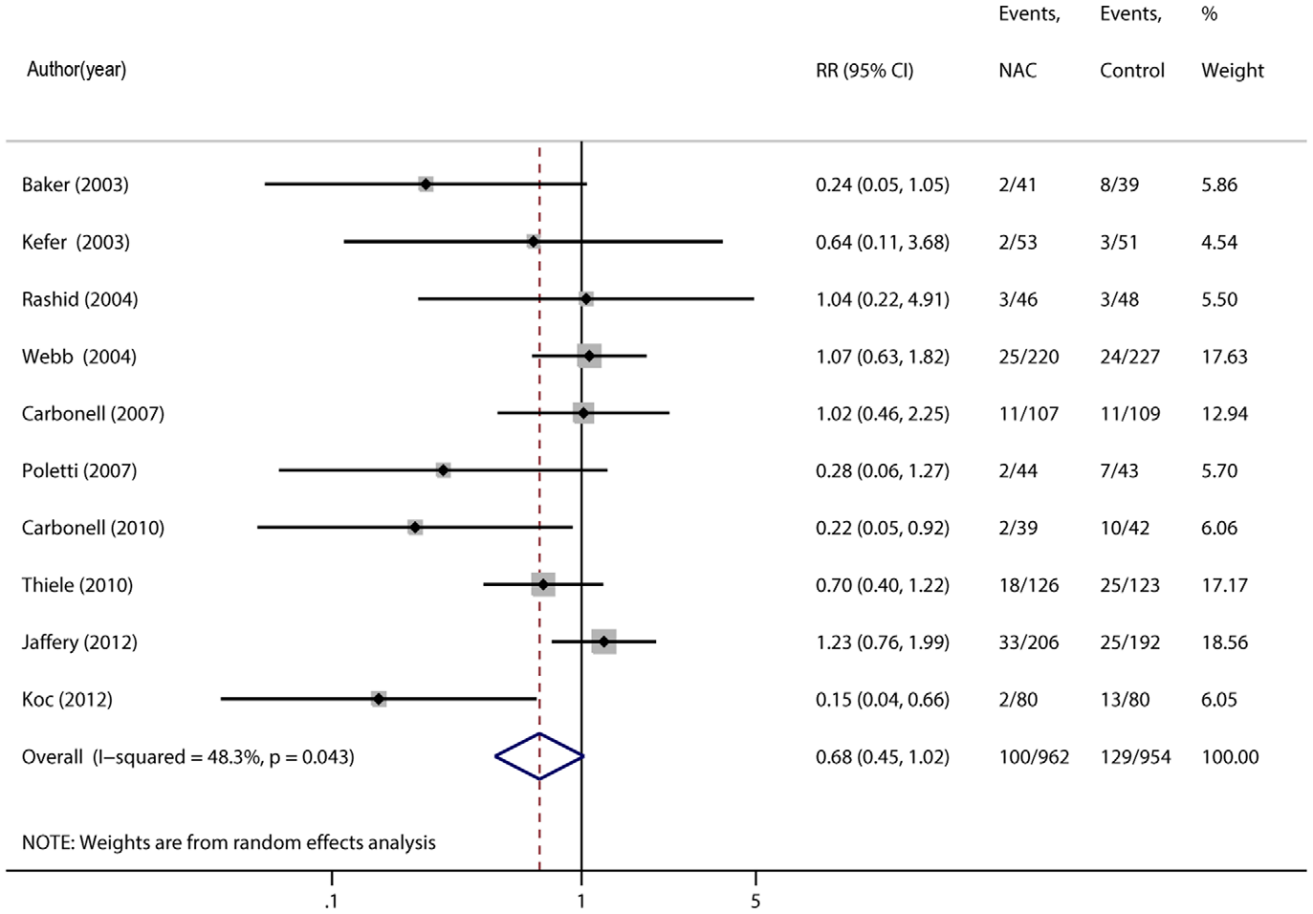
Forest plot of risk ratios (RRs) and 95% confidence intervals (CIs) for the incidence of contrast-induced acute kidney injury in patients assigned to intravenous NAC therapy versus control. Abbreviations: NAC, N-acetylcysteine; RR, risk ratio; CI, confidence interval.

The overall pooled risk ratio (RR) of CIN using a random-effects model was 0.68 (95% CI, 0.45–1.02, p = 0.06) [16]–[25], indicating a nonsignificant trend towards benefit in patients who received NAC (Figure 2). However, there was significant heterogeneity in the analysis comparing the occurrence of CIN across studies (Q = 17.42, P = 0.04; I^2^ = 48%).

### Sensitivity Analyses

We performed several sensitivity analyses. Analyses were repeated in those trials involving cardiac catheterization or peripheral angiography (all studies except Poletti et al. [23]). The summary risk ratio for CIN associated with the use of NAC was essentially unchanged at 0.72 (95% CI, 0.47–1.09, p = 0.12), and substantial heterogeneity remained (P = 0.03). Analyses were also repeated by removing the study by Webb et al. [21] which used a much lower dose (500 mg IV) than the dose used in the other studies. The summary risk ratio did not change substantially but the results became statistically significant (RR = 0.59; 95% CI, 0.36–0.97, p = 0.04), and substantial heterogeneity was again observed (P = 0.03). Finally, restricting our analysis to the studies with a quantified Jadad score of 3 or more demonstrated no benefit for NAC with a summary risk ratio of 0.77 (95% CI, 0.50–1.15, p = 0.20), and no significant heterogeneity was observed (chi-squared test of heterogeneity, P = 0.23).

### Meta-regression

To assess a number of study and patient factors that may have led to heterogeneity between studies, we performed random effects meta-regression examining one covariate at a time (Table 1). These analyses suggested that the heterogeneity may be partially explained by study size with a positive coefficient (coefficient = 0.98, p = 0.08). Other analyses demonstrated that the heterogeneity could not be accounted for by differences in baseline serum creatinine (p = 0.16), volume of contrast media (p = 0.92) or diabetes mellitus (p = 0.83). Likewise, heterogeneity was not accounted for by differences in study quality including use of double-blinding (p = 0.17), intention to treat analysis (p = 0.30), concealment of allocation (p = 0.17) or overall Jadad score (p = 0.59).

**Table 1:** Meta-regression of Possible Sources of Heterogeneity.

Possible Source of Heterogeneity	coefficient*	95% CI	p-value
Baseline Creatinine	−1.02	−2.5 to 0.50	0.16
Contrast volume	−0.08	−1.91 to 1.75	0.92
Diabetes mellitus	−0.01	−0.08 to 0.07	0.83
Total NAC dose	−0.04	−0.20 to 0.12	0.58
Study size	0.98	−0.12 to 2.07	0.08
Publication date	−0.02	−0.18 to 0.15	0.80
Jadad score	0.13	−0.39 to 0.64	0.59
Double-blinding†	0.66	−0.35 to 1.66	0.17
Allocation concealment†	0.73	−0.38 to 1.85	0.17
Intention to treat analysis†	−0.42	−1.67 to 0.82	0.45

* A negative correlation coefficient implies more benefit as the tested independent variable increases.

† For each of these quality components, studies were dichotomized into high or low quality and used through a dummy variable.

### Publication Bias

As a measure of possible publication bias in the primary analysis, an inverted funnel plot was used to explore visually the RR for each study against a measure of its precision (the standard error of the log RR). The funnel plot was asymmetry (Figure S1), which was confirmed by formal statistical testing suggesting the presence of publication bias (Egger’s test, p = 0.013), particularly the absence of small negative trials.

### Secondary Outcomes

#### Renal failure requiring dialysis

The incidence of renal failure requiring dialysis was extremely low, occurred in 7 of the 1914 randomized patients (4 in NAC, 3 in placebo). The pooled risk ratio of renal failure requiring dialysis was 0.72 (95% CI, 0.34 to 4.75, P = 0.72). Heterogeneity was not significant (P = 0.32).

#### Mortality

Complete data on the in-hospital mortality were available from 4 trials [17], [21], [22], [25]. The pooled relative risk of in-hospital mortality with intravenous NAC in the four trials was 0.67 (95% CI, 0.32–1.40, P = 0.29). Significant heterogeneity was not observed (P = 0.99). The study by Carbonell et al. [17] also reported information on 1-year mortality, which was not significantly lower among NAC recipients (15.4% vs. 21.4%, NAC vs. control, respectively, P = 0.67).

#### Length of hospitalization

Only three trials provided information on the length of hospital stay [17], [22], [25] and none of these trials found a significant reduction in length of stay among NAC recipients. There was insufficient data available to be pooled.

#### Adverse events

Specific adverse events were only observed in the study by Baker et al. [16], which found a high rate(14.6%) of transient itching, flushing, and rash among patients receiving the loading dose of 150 mg/kg over 30 minutes. However, these events were mild and safely treated by stopping the therapy and administering hydrocortisone.

## Discussion

This meta-analysis combined results from 10 randomized studies evaluating the effect of intravenous NAC on the incidence of CIN in people receiving intravascular contrast. In our primary analysis, the use of intravenous NAC was associated with a reduction in the incidence of CIN, but this difference was of borderline statistical significance (P = 0.06), and there was significant heterogeneity between trials. In addition, there is insufficient data to show the efficacy of NAC on clinically meaningful endpoints such as dialysis, length of hospital stay or mortality. So the role of intravenous NAC in the prevention of CIN has yet to be defined.

Statistical heterogeneity was present in our analysis. To isolate potential sources of heterogeneity, we conducted a meta-regression that took into account several clinical and study quality factors. Although some baseline characteristics of included patients and some study design details differed between the analysed trials, particularly the mean contrast dose, mean baseline serum creatinine and the proportion of diabetic patients, meta-regression showed no significant relation between these covariates and the relative risk of CIN as a dependent variable. Our meta-regression analysis also explored the potential role of several study quality factors, and none were identified as statistically significant predictors of apparent NAC efficacy across trials. However, meta-regression analyses demonstrated that heterogeneity may be partially explained by study size, with the small studies having the most strongly positive results. As the small studies tended to recruit high-risk patients, at least some of the heterogeneity may be explained by NAC having a greater effect for high-risk patients. An alternative explanation may be that there is publication bias, with small studies that failed to report an effect for NAC treatment not being published.

In sensitivity analysis, because the effect of NAC appeared more homogeneous when only studies with high quality scores were included, it is possible that differences in study quality were responsible for some of the heterogeneity. However, many of these studies did not specify whether or not they fit the quality criteria, with the true quality remaining uncertain.

NAC has antioxidant properties and acts as a vasodilatator. It elevates levels of cyclic guanosine monophosphate and stimulates the release of nitric oxide-derived relaxing factor [43]. The mechanism by which NAC is postulated to be nephroprotective is unclear. Recently, there has been a great increase in interest regarding NAC’s antioxidant properties. Quintavalle et al. [44] have demonstrated that the inhibition of reactive oxygen species (ROS) activation may represent a key mechanism of the protective effect of NAC. However, pharmacokinetic studies have confirmed that only a small proportion of the orally administered NAC enters the systemic circulation in its free form, mainly due to the first-pass metabolism in the liver. Thus, the bioavailability of NAC in individual patients is low [35], [36]. However, a first pass effect after oral administration may allow effective conversion in the liver of acetylcysteine into cysteine and then glutathione, which is a powerful antioxidant with activity against free radicals. It is therefore hypothesized that NAC’s main mechanism of action are mediated through alterations in glutathione metabolism. If this is true, NAC may need to be administered earlier because the oral NAC procedure needs a certain amount of time for NAC to be converted to GSH. However, some animal studies have failed to show a correlation between glutathione levels and renal protection after administration of NAC [45], [46]. The alternative hypothesis is that NAC may exert a direct protective effect on renal cells that have sustained ischemic injury [47], [48]. If this is true, intravenous administration of NAC might be the optimal regimen to be applied, given its rapid onset of effect, higher peak serum NAC levels, and complete bioavailability. Unfortunately, intravenous NAC was not consistently beneficial in the prevention of CIN up to now and there has been a substantial lack of pharmacokinetic or pharmacodynamic components in the trials reported to date, so the exact mechanism by which NAC acts remains unknown.

Intravenous NAC has been assessed for prevention of CIN in contemplation of rapid effect in the situations needed emergency catheterization and also as a result of controversial data obtained from oral pretreatment. There was wide variance in dose of NAC between these studies (from around 7 to 200 mg/kg total doses). Previous studies have provided some evidence for a dose-dependent effect of NAC [36], [49], with more benefit observed when double doses of NAC were administered to reduce CIN. Further, results of a recent meta-analysis indicate that high-dose N-acetylcysteine may decrease the incidence of CIN [50]. Therefore, a study that directly compares the effect of various NAC dose regimens on glomerular filtration rate, renal blood flow, and plasma antioxidant balance might provide the rational selection of NAC regimen for future studies.

In the present studies, the diagnosis of CIN was primarily based on the absolute or relative change in plasma creatinine concentration. However, there has been speculation that NAC may directly decrease sCr without improving GFR, possibly by increasing the metabolism of creatinine or by increasing tubular secretion [51]. It should be noted that this NAC effect has not been demonstrated in patients at high risk for CIN [52], [53]. Nevertheless, serum creatinine may not be an ideal surrogate marker for glomerular filtration rate (GFR), because alterations in renal handling, filtration, secretion and reabsorption may have a profound impact on sCR levels [43], [54]. Furthermore, contrast media themselves may decrease tubular creatinine secretion and thereby leading to a small transient increase in plasma creatinine level, independently of changes in GFR [55]. It has been suggested that newer urinary biomarkers such as cystatin C, KIM-1 or NGAL may be more sensitive to identify kidney damage [56]. However, at present, serum creatinine is the cheapest and most broadly accepted marker of kidney function.

Our meta-analysis has several limitations that should be taken into account. First, the asymmetrical appearance of the funnel plot suggests that publication bias is present, particularly the absence of small studies with negative results. Despite doing a broad search including several international databases and manually searching the conference proceedings and reference lists from identified trials, we cannot rule out that publication bias might lead to an overestimation of the true treatment effect. Although funnel plot asymmetry is often interpreted to indicate publication bias, it is important to consider that this asymmetry may also be due to other sources of bias such as between-study heterogeneity (eg, disparities in the underlying risk of CIN and the intensity of interventions) [34].

Second, meta-regression relies on aggregated data from studies rather than data from individual patients. Therefore, the power to detect a difference in aggregate or to identify explanatory variables using meta-regression is greatly diminished compared with large primary trials with patient-level data. Furthermore, interpretation of any results for study or patient characteristics that must be represented by study population average values or percentages (eg, mean age and percentage with diabetes). Such variables are difficult to model with meta-regression, particularly with a small number of studies. This is known as the ecological fallacy [57]. Thus, meta-regression may fail to find some significant effects. Furthermore, we are unable to assess the impact of hydration on outcome in our meta-regression analyses due to the considerably different hydration protocols among included studies, although some studies have demonstrated that the adoption of hydration may yield an influential efficacy of NAC on the protection of renal functions in patients.

Third, all included studies used the surrogate endpoint of CIN as a primary outcome. Most often this has been defined as an increase in baseline serum creatinine level of 25% or an absolute increase of 44 mmol/L. Despite earlier studies have demonstrated the association of CIN with increased in-hospital morbidity, mortality, and costs of medical care, especially in patients needing dialysis [6], no trial was designed to investigate the effect of NAC on clinically relevant outcomes. Thus, we could not have a sufficient amount of publication data for a meta-analysis to assess the effect of NAC on these relatively rare, but important outcomes.

Finally, studies included in this meta-analysis analyzed the efficacy of NAC with different dose regimens for varied periods of time. It is possible that dose and duration may have differential effect in prevention of CIN. An accepted uniform NAC protocol would ease comparison of clinical and research findings alike.

In conclusion, this meta-analysis showed that research on intravenous N-acetylcysteine and the incidence of CIN is too inconsistent at present to warrant a conclusion on efficacy. In addition, the long-term effect of NAC on more clinically important outcomes has not been established. A large, well designed trial that incorporates the evaluation of clinically relevant outcomes in participants with different underlying risks of CIN is required to more adequately assess the role for intravenous NAC in CIN prevention.

## Supporting Information

Figure S1 **Funnel Plot for Publication Bias.** Funnel plot asymmetry is demonstrated by evidence of a cluster of small studies with low-protective risk and the paucity of small negative studies in the lower right of the funnel plot.
(TIF)

Table S1 **General Characteristics of the 10 Trials.**
(DOC)

Table S2 **Study Quality Characteristics of the 10 Trials.**
(DOC)

Checklist S1 **PRISMA 2009 Checklist.**
(DOC)

Diagram S1 **PRISMA 2009 Flow Diagram.**
(DOC)

## References

[1] Parfrey P (2005) The clinical epidemiology of contrast-induced nephropathy. Cardiovasc Intervent Radiol 28 Suppl 2S3–11. 16419277 10.1007/s00270-005-0196-8

[2] Weisbord SD, Palevsky PM (2005) Radiocontrast-induced acute renal failure. J Intensive Care Med 20: 63–75. 15855219 10.1177/0885066604273503

[3] Nash K, Hafeez A, Hou S (2002) Hospital-acquired renal insufficiency. Am J Kidney Dis 39: 930–936. 11979336 10.1053/ajkd.2002.32766

[4] Briguori C, Manganelli F, Scarpato P, Elia PP, Golia B, et al (2002) Acetylcysteine and contrast agent-associated nephrotoxicity. J Am Coll Cardiol 40: 298–303. 12106935 10.1016/s0735-1097(02)01958-7

[5] Schultz MJ, Baas MC, van der Sluijs HP, Stamkot GA, Smit W (2006) N-acetylcysteine and other preventive measures for contrast-induced nephropathy in the intensive care unit. Curr Med Chem 13: 2565–2570. 17017911 10.2174/092986706778201684

[6] Rihal CS, Textor SC, Grill DE, Berger PB, Ting HH, et al (2002) Incidence and prognostic importance of acute renal failure after percutaneous coronary intervention. Circulation 105: 2259–2264. 12010907 10.1161/01.cir.0000016043.87291.33

[7] Murphy SW, Barrett BJ, Parfrey PS (2000) Contrast nephropathy. J Am Soc Nephrol 11: 177–182. 10616853 10.1681/ASN.V111177

[8] Shalansky SJ, Vu T, Pate GE, Levin A, Humphries KH, et al (2005) N-acetylcysteine for prevention of radiographic contrast material-induced nephropathy: is the intravenous route best? Pharmacotherapy 25: 1095–1103. 16207100 10.1592/phco.2005.25.8.1095

[9] Tepel M, van der Giet M, Schwarzfeld C, Laufer U, Liermann D, et al (2000) Prevention of radiographic-contrast-agent-induced reductions in renal function by acetylcysteine. N Engl J Med 343: 180–184. 10900277 10.1056/NEJM200007203430304

[10] Stacul F, van der Molen A, Reimer P, Webb J, Thomsen H, et al.. (2011) Contrast induced nephropathy: updated ESUR Contrast Media Safety Committee guidelines. 2527–2541. 10.1007/s00330-011-2225-0 21866433

[11] Olsson B, Johansson M, Gabrielsson J, Bolme P (1988) Pharmacokinetics and bioavailability of reduced and oxidized N-acetylcysteine. Eur J Clin Pharmacol 34: 77–82. 3360052 10.1007/BF01061422

[12] Borgstrom L, Kagedal B, Paulsen O (1986) Pharmacokinetics of N-acetylcysteine in man. Eur J Clin Pharmacol 31: 217–222. 3803419 10.1007/BF00606662

[13] Tsikas D, Sandmann J, Ikic M, Fauler J, Stichtenoth DO, et al (1998) Analysis of cysteine and N-acetylcysteine in human plasma by high-performance liquid chromatography at the basal state and after oral administration of N-acetylcysteine. J Chromatogr B Biomed Sci Appl 708: 55–60. 9653946 10.1016/s0378-4347(97)00670-1

[14] Burgunder JM, Varriale A, Lauterburg BH (1989) Effect of N-acetylcysteine on plasma cysteine and glutathione following paracetamol administration. Eur J Clin Pharmacol 36: 127–131. 2721538 10.1007/BF00609183

[15] Shalansky SJ, Pate GE, Levin A, Webb JG (2005) N-acetylcysteine for prevention of radiocontrast induced nephrotoxicity: the importance of dose and route of administration. Heart 91: 997–999. 16020580 10.1136/hrt.2004.053579 PMC1769030

[16] Baker CS, Wragg A, Kumar S, De Palma R, Baker LR, et al (2003) A rapid protocol for the prevention of contrast-induced renal dysfunction: the RAPPID study. J Am Coll Cardiol 41: 2114–2118. 12821233 10.1016/s0735-1097(03)00487-x

[17] Carbonell N, Sanjuan R, Blasco M, Jorda A, Miguel A (2010) N-acetylcysteine: short-term clinical benefits after coronary angiography in high-risk renal patients. Rev Esp Cardiol 63: 12–19. 10.1016/s1885-5857(10)70004-9 20089221

[18] Koc F, Ozdemir K, Kaya MG, Dogdu O, Vatankulu MA, et al (2012) Intravenous N-acetylcysteine plus high-dose hydration versus high-dose hydration and standard hydration for the prevention of contrast-induced nephropathy: CASIS–a multicenter prospective controlled trial. Int J Cardiol 155: 418–423. 21106264 10.1016/j.ijcard.2010.10.041

[19] Kefer JM, Hanet CE, Boitte S, Wilmotte L, De Kock M (2003) Acetylcysteine, coronary procedure and prevention of contrast-induced worsening of renal function: which benefit for which patient? Acta Cardiol 58: 555–560. 14713182 10.2143/AC.58.6.2005321

[20] Rashid ST, Salman M, Myint F, Baker DM, Agarwal S, et al (2004) Prevention of contrast-induced nephropathy in vascular patients undergoing angiography: a randomized controlled trial of intravenous N-acetylcysteine. J Vasc Surg 40: 1136–1141. 15622367 10.1016/j.jvs.2004.09.026

[21] Webb JG, Pate GE, Humphries KH, Buller CE, Shalansky S, et al (2004) A randomized controlled trial of intravenous N-acetylcysteine for the prevention of contrast-induced nephropathy after cardiac catheterization: lack of effect. Am Heart J 148: 422–429. 15389228 10.1016/j.ahj.2004.03.041

[22] Carbonell N, Blasco M, Sanjuan R, Perez-Sancho E, Sanchis J, et al (2007) Intravenous N-acetylcysteine for preventing contrast-induced nephropathy: a randomised trial. Int J Cardiol 115: 57–62. 16814414 10.1016/j.ijcard.2006.04.023

[23] Poletti PA, Saudan P, Platon A, Mermillod B, Sautter AM, et al (2007) I.v. N-acetylcysteine and emergency CT: use of serum creatinine and cystatin C as markers of radiocontrast nephrotoxicity. AJR Am J Roentgenol 189: 687–692. 17715118 10.2214/AJR.07.2356

[24] Thiele H, Hildebrand L, Schirdewahn C, Eitel I, Adams V, et al (2010) Impact of high-dose N-acetylcysteine versus placebo on contrast-induced nephropathy and myocardial reperfusion injury in unselected patients with ST-segment elevation myocardial infarction undergoing primary percutaneous coronary intervention. The LIPSIA-N-ACC (Prospective, Single-Blind, Placebo-Controlled, Randomized Leipzig Immediate PercutaneouS Coronary Intervention Acute Myocardial Infarction N-ACC) Trial. J Am Coll Cardiol 55: 2201–2209. 20466200 10.1016/j.jacc.2009.08.091

[25] Jaffery Z, Verma A, White CJ, Grant AG, Collins TJ, et al (2012) A randomized trial of intravenous N-acetylcysteine to prevent contrast induced nephropathy in acute coronary syndromes. Catheter Cardiovasc Interv 79: 921–926. 21542122 10.1002/ccd.23157

[26] Moher D, Liberati A, Tetzlaff J, Altman DG (2009) Preferred reporting items for systematic reviews and meta-analyses: the PRISMA statement. PLoS Med 6: e1000097. 19621072 10.1371/journal.pmed.1000097 PMC2707599

[27] Molitoris BA, Levin A, Warnock DG, Joannidis M, Mehta RL, et al (2007) Improving outcomes of acute kidney injury: report of an initiative. Nat Clin Pract Nephrol 3: 439–442. 17653122 10.1038/ncpneph0551

[28] Verhagen AP, de Vet HC, de Bie RA, Kessels AG, Boers M, et al (1998) The Delphi list: a criteria list for quality assessment of randomized clinical trials for conducting systematic reviews developed by Delphi consensus. J Clin Epidemiol 51: 1235–1241. 10086815 10.1016/s0895-4356(98)00131-0

[29] Jadad AR, Moore RA, Carroll D, Jenkinson C, Reynolds DJ, et al (1996) Assessing the quality of reports of randomized clinical trials: is blinding necessary? Control Clin Trials 17: 1–12. 8721797 10.1016/0197-2456(95)00134-4

[30] Thompson SG, Sharp SJ (1999) Explaining heterogeneity in meta-analysis: a comparison of methods. Stat Med 18: 2693–2708. 10521860 10.1002/(sici)1097-0258(19991030)18:20<2693::aid-sim235>3.0.co;2-v

[31] Higgins JP, Thompson SG, Deeks JJ, Altman DG (2003) Measuring inconsistency in meta-analyses. BMJ 327: 557–560. 12958120 10.1136/bmj.327.7414.557 PMC192859

[32] DerSimonian R, Laird N (1986) Meta-analysis in clinical trials. Control Clin Trials 7: 177–188. 3802833 10.1016/0197-2456(86)90046-2

[33] Thompson SG, Higgins JP (2002) How should meta-regression analyses be undertaken and interpreted? Stat Med 21: 1559–1573. 12111920 10.1002/sim.1187

[34] Sterne JA, Egger M, Smith GD (2001) Systematic reviews in health care: Investigating and dealing with publication and other biases in meta-analysis. BMJ 323: 101–105. 11451790 10.1136/bmj.323.7304.101 PMC1120714

[35] Egger M, Davey Smith G, Schneider M, Minder C (1997) Bias in meta-analysis detected by a simple, graphical test. BMJ 315: 629–634. 9310563 10.1136/bmj.315.7109.629 PMC2127453

[36] Marenzi G, Assanelli E, Marana I, Lauri G, Campodonico J, et al (2006) N-acetylcysteine and contrast-induced nephropathy in primary angioplasty. N Engl J Med 354: 2773–2782. 16807414 10.1056/NEJMoa054209

[37] Aslanger E, Uslu B, Akdeniz C, Polat N, Cizgici Y, et al (2012) Intrarenal application of N-acetylcysteine for the prevention of contrast medium-induced nephropathy in primary angioplasty. Coron Artery Dis 23: 265–270. 22343798 10.1097/MCA.0b013e328351aacc

[38] Ratcliffe JA, Thiagarajah P, Chen J, Kavala G, Kanei Y, et al (2009) Prevention of contrast-induced nephropathy: A randomized controlled trial of sodium bicarbonate and N-acetylcysteine. Int J Angiol 18: 193–197. 22477552 10.1055/s-0031-1278353 PMC2903033

[39] Recio-Mayoral A, Chaparro M, Prado B, Cozar R, Mendez I, et al (2007) The reno-protective effect of hydration with sodium bicarbonate plus N-acetylcysteine in patients undergoing emergency percutaneous coronary intervention: the RENO Study. J Am Coll Cardiol 49: 1283–1288. 17394959 10.1016/j.jacc.2006.11.034

[40] Baranska-Kosakowska A, Zakliczynski M, Przybylski R, Zembala M (2007) Role of N-acetylcysteine on renal function in patients after orthotopic heart transplantation undergoing coronary angiography. Transplant Proc 39: 2853–2855. 18022000 10.1016/j.transproceed.2007.08.057

[41] Briguori C, Airoldi F, D'Andrea D, Bonizzoni E, Morici N, et al (2007) Renal Insufficiency Following Contrast Media Administration Trial (REMEDIAL): a randomized comparison of 3 preventive strategies. Circulation 115: 1211–1217. 17309916 10.1161/CIRCULATIONAHA.106.687152

[42] Kotlyar E, Keogh AM, Thavapalachandran S, Allada CS, Sharp J, et al (2005) Prehydration alone is sufficient to prevent contrast-induced nephropathy after day-only angiography procedures–a randomised controlled trial. Heart Lung Circ 14: 245–251. 16360994 10.1016/j.hlc.2005.06.007

[43] Kiefer P, Vogt J, Radermacher P (2000) From mucolytic to antioxidant and liver protection: new aspects in the intensive care unit career of N-acetylcysteine. Crit Care Med 28: 3935–3936. 11153640 10.1097/00003246-200012000-00037

[44] Quintavalle C, Brenca M, De Micco F, Fiore D, Romano S, et al (2011) In vivo and in vitro assessment of pathways involved in contrast media-induced renal cells apoptosis. Cell Death Dis 2: e155. 21562587 10.1038/cddis.2011.38 PMC3122117

[45] Heyman SN, Goldfarb M, Shina A, Karmeli F, Rosen S (2003) N-acetylcysteine ameliorates renal microcirculation: studies in rats. Kidney Int 63: 634–641. 12631128 10.1046/j.1523-1755.2003.00783.x

[46] Weinbroum AA, Rudick V, Ben-Abraham R, Karchevski E (2000) N-acetyl-L-cysteine for preventing lung reperfusion injury after liver ischemia-reperfusion: a possible dual protective mechanism in a dose-response study. Transplantation 69: 853–859. 10755539 10.1097/00007890-200003150-00031

[47] DiMari J, Megyesi J, Udvarhelyi N, Price P, Davis R, et al (1997) N-acetyl cysteine ameliorates ischemic renal failure. Am J Physiol 272: F292–298. 9087670 10.1152/ajprenal.1997.272.3.F292

[48] Hashimoto S, Gon Y, Matsumoto K, Takeshita I, Horie T (2001) N-acetylcysteine attenuates TNF-alpha-induced p38 MAP kinase activation and p38 MAP kinase-mediated IL-8 production by human pulmonary vascular endothelial cells. Br J Pharmacol 132: 270–276. 11156586 10.1038/sj.bjp.0703787 PMC1572545

[49] Briguori C, Colombo A, Violante A, Balestrieri P, Manganelli F, et al (2004) Standard vs double dose of N-acetylcysteine to prevent contrast agent associated nephrotoxicity. Eur Heart J 25: 206–211. 14972420 10.1016/j.ehj.2003.11.016

[50] Trivedi H, Daram S, Szabo A, Bartorelli AL, Marenzi G (2009) High-dose N-acetylcysteine for the prevention of contrast-induced nephropathy. Am J Med 122: 874 e879–815. 10.1016/j.amjmed.2009.01.035 19699385

[51] Hoffmann U, Fischereder M, Kruger B, Drobnik W, Kramer BK (2004) The value of N-acetylcysteine in the prevention of radiocontrast agent-induced nephropathy seems questionable. J Am Soc Nephrol 15: 407–410. 14747387 10.1097/01.asn.0000106780.14856.55

[52] Haase M, Haase-Fielitz A, Ratnaike S, Reade MC, Bagshaw SM, et al (2008) N-Acetylcysteine does not artifactually lower plasma creatinine concentration. Nephrol Dial Transplant 23: 1581–1587. 18202091 10.1093/ndt/gfm818

[53] Moist L, Sontrop JM, Gallo K, Mainra R, Cutler M, et al (2010) Effect of N-acetylcysteine on serum creatinine and kidney function: results of a randomized controlled trial. Am J Kidney Dis 56: 643–650. 20541301 10.1053/j.ajkd.2010.03.028

[54] Walser M (1998) Assessing renal function from creatinine measurements in adults with chronic renal failure. Am J Kidney Dis 32: 23–31. 9669420 10.1053/ajkd.1998.v32.pm9669420

[55] Brautigam M, Persson PB (2006) Do iodinated contrast media interfere with renal tubular creatinine secretion? Radiology 240: 615; author reply 615. 10.1148/radiol.2402051532 16864687

[56] Ariarajah N, Gerstel E, Martin PY, Ponte B (2011) [Biomarkers in acute kidney injury: an update]. Rev Med Suisse 7: 490–494. 21462518

[57] Schmid CH, Stark PC, Berlin JA, Landais P, Lau J (2004) Meta-regression detected associations between heterogeneous treatment effects and study-level, but not patient-level, factors. J Clin Epidemiol 57: 683–697. 15358396 10.1016/j.jclinepi.2003.12.001

