# Supplementary figures and images for: Intravenous N-Acetylcysteine for Prevention of Contrast-Induced Nephropathy: A Meta-Analysis of Randomized, Controlled Trials
Source: PLoS One. 2013 Jan 30;8(1):e55124. doi: 10.1371/journal.pone.0055124 (PMC3559541; doi:10.1371/journal.pone.0055124)

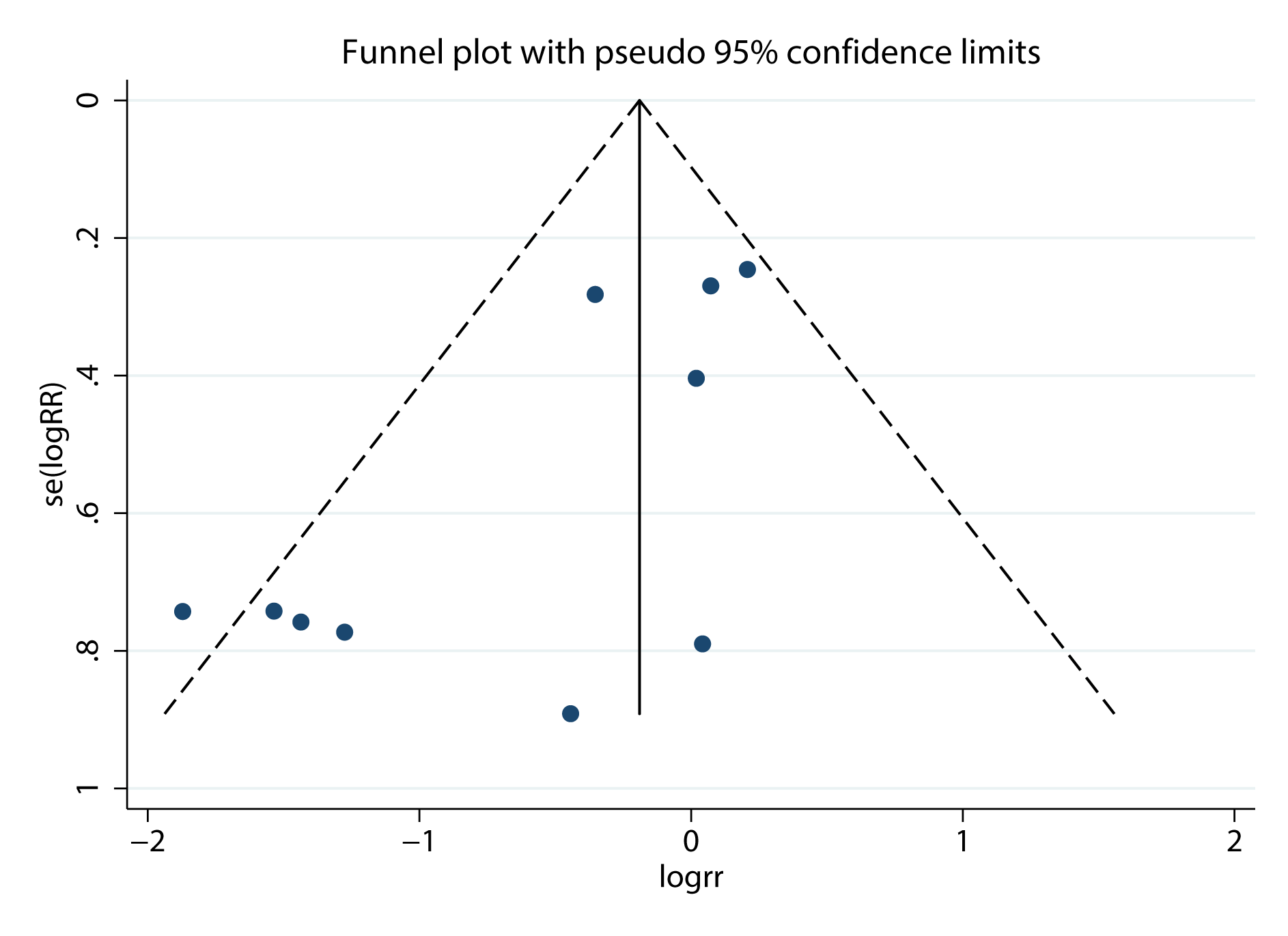

Supplement: Figure S1 — Funnel Plot for Publication Bias. Funnel plot asymmetry is demonstrated by evidence of a cluster of small studies with low-protective risk and the paucity of small negative studies in the lower right of the funnel plot. (TIF) [file pone.0055124.s001.tif]
