# Supplementary material for: Intravenous N-Acetylcysteine for Prevention of Contrast-Induced Nephropathy: A Meta-Analysis of Randomized, Controlled Trials
Source: PLoS One. 2013 Jan 30;8(1):e55124. doi: 10.1371/journal.pone.0055124 (PMC3559541; doi:10.1371/journal.pone.0055124)
Supplement: Table S1 — General Characteristics of the 10 Trials. (DOC) [file pone.0055124.s002.doc]

**Table 1. General Characteristics of the 1**0 Trials

| **Authors (year)** | **Contrast procedure** | **Renal Function**  **Defining Inclusion** | **Definition of CIN**  **(SCr rise)** | **Time of Definition** | **Contrast Agent** | **Average Contrast  Volume**  **(mL)** | **Hydration**  **regimen** | **NAC  regimen** | **Cumulative NAC Dose (mg)** | **Male**  **(%)** | **Diabetes Mellitus**  **(%)** | **Baseline  Serum Creatinine**  **(mg/dL)** | **Overall Incidence  of CIN(%)** |
| --- | --- | --- | --- | --- | --- | --- | --- | --- | --- | --- | --- | --- | --- |
| **Baker**  **(2003)** | CC | Cr>1.36 or CrCl<50 | ≥25% | 96 h | Iodixanol | 230.2 | 0.9%NS 1 mL/kg/hr 12 hr pre/post in control arm* | 150 mg/kg IV 30 min pre & 50 mg/kg IV infusion 4 hr post | 200 per kg | 87.5 | 42.5 | 1.8 | 12.5 |
| **Kefer (2003)** | CC | Cr ≤ 3 | ≥25% or ≥0.5 mg/dl | 24 h | Iopromide  or iohexol | 199 | D5W 20 mL/hr 12 hr pre & 24 hr post | 1200 mg in 0.9% NS IV over 60 min 12 h pre & immediately post | 2400 | 76.9 | 12.5 | 1.1 | 4.8 |
| **Rashid**  **(2004)** | PA | No minimum Cr  required | ≥25% or ≥0.5 mg/dl | 48 h | Iohexol | 143.5 | 0.9%NS 500 mL over 6 to 12 hrs pre/post | 1000 mg in 0.9% NS IV 6 to 12 hrs pre/post | 2000 | 63.8 | 31.9 | 1.33 | 6.4 |
| **Webb (2004)** | CC | CrCl<50 | 0.5 mg/dl | Within 7 d | ioversol | 120 | 0.9% NS 200 mL pre &1.5 mL/kg/h 6 h or discharge(<6h) post | 500 mg in D5NS IV over 15 min, 1 h pre | 500 | 60.8 | 34.9 | 1.6 | 6.5 |
| **Carbonell(2007)** | CC | Normal renal function | >25% or ≥0.5 mg/d | 48 h | Iopromide | 188 | 0.45% NS 1 mL/kg/h at least 6 h pre & 12 h post | 600 mg in 50ml of 0.9%NS IV over 30min bid ×2 d, at least one dose 6h pre | 2400 | 76.4 | 33.3 | 0.95 | 10.2 |
| **Poletti**  **(2007)** | CT scan | Cr>1.2 | ≥ 25% | 48 h | Iopromide | 125.5 | 0.45% NS 5 mL/kg 1h pre & 1 mL/kg/h 12 h post | 900 mg in 50ml of D5W IV 1h pre & 900mg in 0.45% NS 1 mL/kg/h 12 h post | 1800 | 63.2 | 17.2 | 1.66 | 15.6 |
| **Carbonell**  **(2010)** | CC | Cr ≥1.4 or CrCl<50 | >25% or ≥0.5 mg/d | 48 h | iopromide | 160.6 | 0.45% NS 1 mL/kg/h 6 h pre & 12 h post | 600 mg in 50 mL of 0.9% NS IV over 30min bid ×2 d | 2400 | 80.2 | 46.9 | 1.94 | 14.8 |
| **Thiele**  **(2010)** | PCI | No minimum Cr  required | ≥25% | 24-72h | iopromide | 170 | 0.9%NS 1 mL/kg/h 12 hr post | 1200 mg IV bolus pre & 1200 mg IV bid × 2 d post | 6000 | 68.1 | 29.1 | 0.90 | 17.3 |
| **Jaffery**  **(2012)** | CC | No minimum Cr  required | ≥ 25% | 48-72 h | Iodixanol | 165.5 | 500 ml of 5% dextrose + 0.9%NS  1 mL/kg/h 24 hr | 1200 mg IV bolus and followed by 200 mg/h for the next 24 h | 6000 | 63.3 | 34.4 | 1.08 | 14.6 |
| **Koc**  **(2012) †** | CC | Cr≥1.1or CrCl≤60 | ≥25% or ≥0.5 mg/d | 48 h | iohexol | 125 | 0.9%NS 1 mL/kg/h 24 h pre/post | 600 mg IV bid× 2 d, before and on the day of procedure | 2400 | 77.5 | 31.9 | 1.3 | 9.4 |

**Abbreviations: sCr, serum creatinine (mg/dL); CrCl, creatinine clearance (mL/min); CC, cardiac catheterization; CT, computed tomography; PA, peripheral angiography; IV, intravenous; NAC, N-acetylcysteine; D5NS, dextrose 5%in normal saline**

***NAC recipients received 500 mL of intravenous saline over 30 mins before the procedure, and 500 mL over 4-hour post-procedure.**

**† This trial had three experimental arms: NAC plus high-dose hydration, high-dose hydration, and standard hydration. Table considers only the NAC and high-dose hydration arms.**
