# Supplementary material for: Intravenous N-Acetylcysteine for Prevention of Contrast-Induced Nephropathy: A Meta-Analysis of Randomized, Controlled Trials
Source: PLoS One. 2013 Jan 30;8(1):e55124. doi: 10.1371/journal.pone.0055124 (PMC3559541; doi:10.1371/journal.pone.0055124)
Supplement: Table S2 — Study Quality Characteristics of the 10 Trials. (DOC) [file pone.0055124.s003.doc]

Table 2. Study Quality Characteristics

| Author,  (Year) | Jadad Score | Randomi-  Zation  process described | Similar  Baseline | Inclusion/  exclusion  criteria  specified | Placebo  Controlled | Concealment  of  Allocation | Extent of  blinding | Intention  to treat  analysis | Power  calculation | Reported  loss to  follow-up |
| --- | --- | --- | --- | --- | --- | --- | --- | --- | --- | --- |
| Baker(2003) | 2 | No | Yes | Yes/Yes | No | NS | NS | Yes | Yes | Yes |
| Kefer (2003) | 2 | Yes | Yes | Yes/Yes | Yes | NS | Single | No | No | Yes |
| Rashid (2004) | 3 | Yes | Yes | Yes/No | Yes | Yes | Double | No | Yes | Yes |
| Webb (2004) | 5 | Yes | Yes | Yes/Yes | Yes | Yes | Double | Yes | Yes | Yes |
| Carbonell(2007) | 4 | Yes | Yes | Yes/Yes | Yes | Yes | Double | No | No | Yes |
| Poletti(2007) | 3 | No | Yes | Yes/Yes | Yes | No | Double | No | No | Yes |
| Carbonell(2010) | 4 | Yes | Yes | Yes/Yes | Yes | No | Double | Yes | No | Yes |
| Thiele(2010) | 3 | Yes | Yes | Yes/Yes | Yes | No | Single | No | No | Yes |
| Jaffery(2012) | 2 | No | Yes | Yes/Yes | Yes | No | Double | No | Yes | Yes |
| Koc(2012) | 2 | No | Yes | Yes/Yes | No | No | NS | No | Yes | Yes |

Abbreviation: NS, not specified or available.
