# Supplementary material for: Intravenous N-Acetylcysteine for Prevention of Contrast-Induced Nephropathy: A Meta-Analysis of Randomized, Controlled Trials
Source: PLoS One. 2013 Jan 30;8(1):e55124. doi: 10.1371/journal.pone.0055124 (PMC3559541; doi:10.1371/journal.pone.0055124)
Supplement: Diagram S1 — PRISMA 2009 Flow Diagram. (DOC) [file pone.0055124.s005.doc]

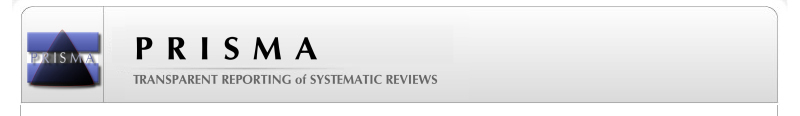
**PRISMA 2009 Flow Diagram**

**Screening**

**Included**

**Eligibility**

**Identification**

Search results screened (n=476)

Potentially relevant publications screened by abstracts (n=289)

Potentially relevant RCTs for full-text review (n=17)

Potentially appropriate RCTs to be included in meta-analysis (n=11)

10 RCTs included in final analysis

Excluded duplicates (n=187)

Excluded by abstracts (n=272)

Reason: NAC administered orally, irrelevant topic, review articles, nonhuman trials, letters, trial designs or posters.

RCTs excluded (n=1)

Reason: no CINs observed in both treatment and control group.

RCTs excluded (n=6)

Reason: NAC administered intravenously plus orally (n=3), combined NAC with other antioxidants (n=1), CIN not defined (n=1), no control group (n=1).
